# Daily preventive zinc supplementation increases the antibody response against pathogenic *Escherichia coli* in children with zinc insufficiency: a randomised controlled trial

**DOI:** 10.1038/s41598-022-20445-8

**Published:** 2022-09-27

**Authors:** Chidchamai Kewcharoenwong, Myint Myint Sein, Arnone Nithichanon, Aranya Khongmee, K Ryan Wessells, Guy-Marino Hinnouho, Maxwell A. Barffour, Sengchanh Kounnavong, Sonja Y. Hess, Charles B. Stephensen, Ganjana Lertmemongkolchai

**Affiliations:** 1 Faculty of Associated Medical Sciences, Chiang Mai University, Chiang Mai, 50200 Thailand; 2 The Centre for Research & Development of Medical Diagnostic Laboratories, Faculty of Associated Medical Sciences, Khon Kaen University, Khon Kaen, Thailand; 3 Department of Microbiology, Faculty of Medicine, Research and Diagnostic Center for Emerging Infectious Diseases (RCEID), Khon Kaen University, Khon Kaen, Thailand; 4 Department of Nutrition, Institute for Global Nutrition, University of California, Davis, CA USA; 5 Helen Keller International, Washington, DC USA; 6 Public Health Program, College of Health and Human Services, Missouri State University, Springfield, MO USA; 7 Lao Tropical and Public Health Institute, Vientiane, Lao People’s Democratic Republic; 8 Agricultural Research Service, Western Human Nutrition Research Center, USDA, Davis, CA USA

**Keywords:** Immunology, Medical research

## Abstract

Zinc deficiency impairs the antibody-mediated immune response and is common in children from lower-income countries. This study aimed to investigate the impact of different zinc supplementation regimens (7, 10 or 20 mg/day elemental zinc)—therapeutic dispersible zinc tablets (TZ), daily multiple micronutrient powder (MNP), daily preventive zinc tablets (PZ) and placebo powder (control)—and compare between baseline and endline antibody production against pathogenic *Escherichia coli* in Laotian children (aged 6–23 months). Fifty representative plasma samples of each treatment group were randomly selected from 512 children to determine anti-*E. coli* IgG antibody levels and avidity. Of the 200 children, 78.5% had zinc deficiency (plasma zinc concentration < 65 µg/dL) and 40% had anaemia before receiving zinc supplementation. aAfter receiving the TZ, MNP or PZ regimen, the plasma anti-*E. coli* IgG levels were significantly increased compared with baseline; the effect on the antibody level was more pronounced in children with zinc deficiency. Interestingly, there was increased anti-*E. coli* IgG avidity in the control and PZ groups. This study suggests that PZ might be the optimal zinc supplementation regimen to increase both the quantity and quality of antibody responses in children with zinc deficiency. Clinical trial registration: https://clinicaltrials.gov/ct2/show/NCT02428647 (NCT02428647, 29/04/2015).

## Introduction

Zinc is an essential micronutrient to maintain regular biological maturation, neurocognitive development as well as immune function^1^. Immune response modulation by zinc has been reported via release of glucocorticoids, decreased thymulin and antioxidant activity^2^. Dysregulation of zinc homeostasis affects adaptive immune responses and causes immunodeficiency^3^. A major component of the adaptive immune system is the humoral immune response, also called the antibody-mediated immune response^4^. Binding of zinc to SLC39A10/ZIP10 or zinc transporter modulates the B-cell receptor (BCR) signal strength, resulting in the induction of an antibody-mediated immune response^5^.

The prevalence of zinc deficiency is estimated to range from 7.5% in high-income regions to 30% in South Asia^6^. Multiple systematic reviews have reported that preventive zinc supplementation is associated with a decrease in diarrhoea- and pneumonia-related morbidity and mortality in children in lower-income countries^7,8^. Diarrhoeal disease is the second leading cause of death in children under 5 years old^9^. In those areas, pathogenic *Escherichia coli* strains and *Salmonella* are commonly found in children with persistent diarrhoea^10^. Moreover, sepsis is present in about 22.5% of children with diarrhoea; it is cause by the translocation of gram-negative bacteria through the diseased and inflamed gut^11^. Antibodies, especially immunoglobulin G (IgG), encounter and neutralise the bacteria and their toxins^12,13^. Antibody avidity has been used as a measure of functional maturation of the humoral immune response, and increases in antibody avidity over time have been shown after both infection and vaccination^14,15^.

A previous randomised controlled trial investigated zinc supplementation in 512 rural Laotian children. There were four intervention groups: (1) daily placebo (Control), (2) therapeutic dispersible zinc tablets (TZ) as part of 10-day treatment of diarrhoea, (3) daily multiple micronutrient powder including zinc (MNP) and (4) daily preventive zinc tablets (PZ). Children aged 6–23 months were randomly assigned to one of these interventions in a community-based intervention trial for approximately 9 months. The parent studies examined child growth, diarrhoeal morbidity and the haematologic and micronutrient statuses^16–23^. PZ and MNP supplementation significantly increased the plasma zinc concentration compared with the control and TZ groups, but there was no impact on growth or overall diarrhoea burden^19^. Interestingly, the previous sub-study on the immune response found that zinc supplementation, especially PZ supplementation, decreased lymphocyte and eosinophil concentrations, although there was no effect on cytokine concentrations or T cell levels^17^. However, the humoral antibody response of this trial has not yet been reported.

This report represents a sub-study to investigate the impact of zinc supplementation on antibody production against pathogenic *E. coli* assayed in surplus aliquots of plasma samples from the parent trial in Laotian children. Plasma IgG levels and the avidity against pathogenic *E. coli* were quantified by enzyme-linked immunosorbent assay (ELISA) and analysed with respect to the zinc status at baseline. TZ, MNP and PZ could increase the plasma IgG level, but only PZ could improve the avidity of anti-*E. coli* antibodies.

## Results

### Demographic characteristics, zinc status and complete blood count (CBC) data

At baseline, the mean ± standard deviation (SD) age of children was 15.1 ± 5.4 months and 56.5% of them were male (Table 1). The mean ± SD plasma zinc concentration was 56.1 ± 12.7 μg/dL, with 78.5% zinc deficient based on the cut-off of 65 μg/dL^24^. The mean ± SD haemoglobin (Hb) concentration was 11.1 ± 0.9 g/dL with 40.0% of children at < 11 g/dL (lower limit of normal Hb in children aged 0.5–4 years^25^), suggesting that they had anaemia prior to the intervention. According to the Nakhonphanom Hospital’s reference ranges, 2.5% of children had increased white blood cells (range 4–11 × 10^3^ cells/µL) with increased percentages of neutrophils (range 50–70%), lymphocytes (range 20–40%), monocytes (range 0–7%), basophils (range 0–1%) and eosinophils (range 0–1%), indicating inflammation or infection.

**Table 1:** Baseline characteristics of the total study population (All) and each intervention group.

Characteristic	All (n = 200)	Control (n = 50)	TZ (n = 50)	MNP (n = 50)	PZ (n = 50)
Age, month(s)	15.1 ± 5.4	14.4 ± 5.7	15.2 ± 5.2	15.5 ± 5.3	15.3 ± 5.4
Males	56.5 (113)	62.0 (31)	46.0 (23)	58.0 (29)	61.2 (30)
Plasma zinc, µg/dL	56.1 ± 12.7	56.1 ± 12.3	57.6 ± 12.4	54.3 ± 10.0	56.5 ± 15.7
Hb, g/dL	11.1 ± 0.9	11.1 ± 0.8	11.1 ± 0.9	11.1 ± 0.8	11.1 ± 0.9
White blood cells, × 10^3^/µL	11.4 ± 3.2	11.5 ± 3.2	11.2 ± 3.2	11.4 ± 2.7	11.4 ± 3.6
Neutrophils, %	27.8 ± 11.8	28.0 ± 12.1	28.3 ± 11.9	27.6 ± 11.5	27.1 ± 11.8
Lymphocytes, %	55.9 ± 13.2	57.6 ± 13.2	55.2 ± 13.1	55.0 ± 14.0	55.9 ± 12.8
Monocytes, %	5.8 ± 3.8	5.8 ± 3.2	5.9 ± 3.7	5.4 ± 3.1	6.3 ± 5.1
Eosinophils, %	4.0 ± 4.2	3.5 ± 3.6	4.0 ± 3.4	4.0 ± 3.7	4.6 ± 5.7
Basophils, %	0.4 ± 0.6	0.3 ± 0.4	0.5 ± 0.7	0.3 ± 0.4	0.5 ± 0.7

The values are the mean ± standard deviation or % (n).

Control, placebo; *TZ* therapeutic dispersible zinc tablets, *MNP* daily multiple micronutrient powder, *PZ* daily preventive zinc tablets, *Hb* haemoglobin.

### Plasma IgG levels in response to pathogenic *E. coli*

**Figure 1:**
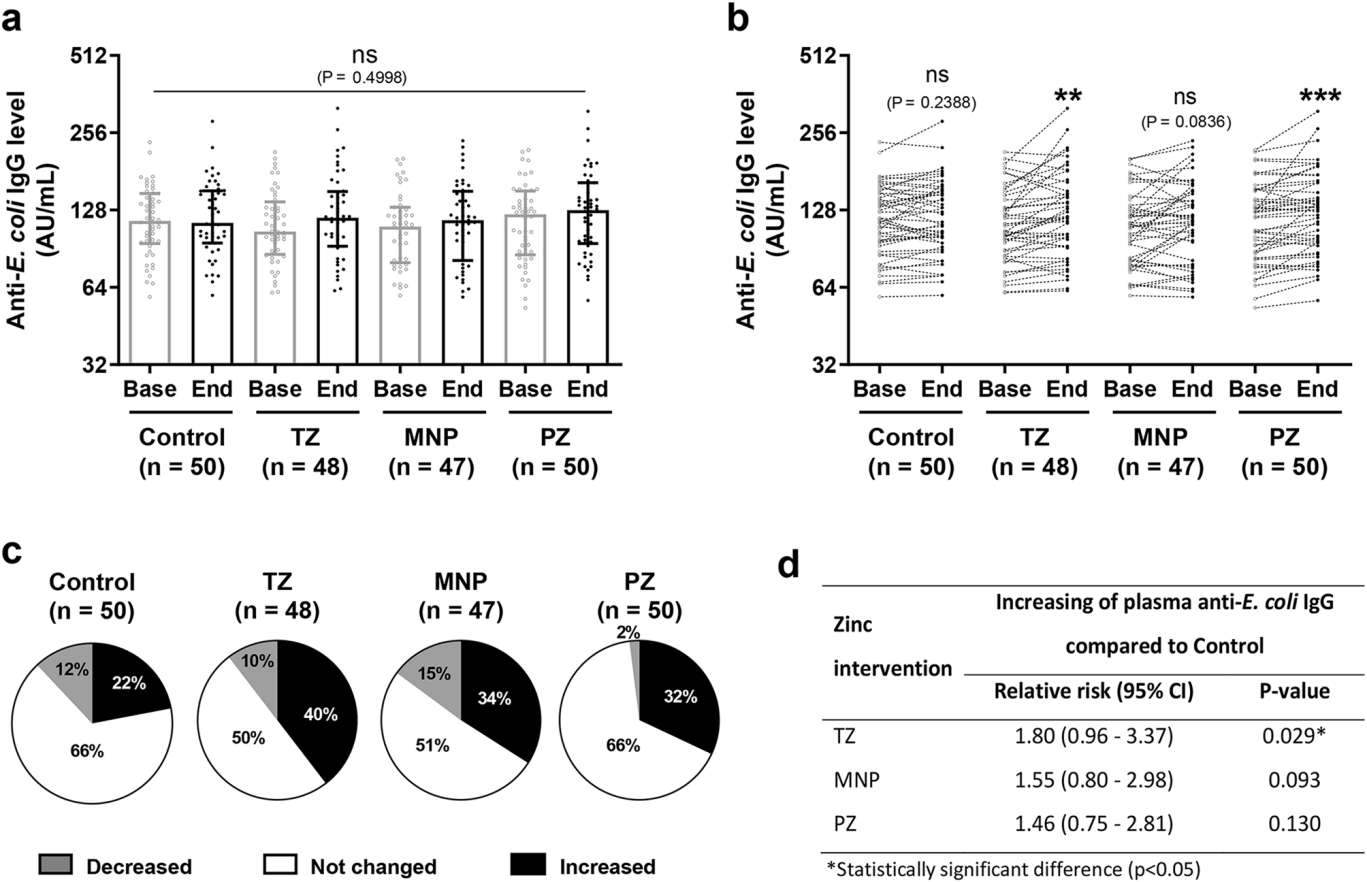
Plasma IgG levels in response to pathogenic *Escherichia coli* of the four intervention groups. (**a**) Plasma samples from baseline and endline of each intervention group (Control, n = 50; TZ, therapeutic zinc with diarrhoea, n = 48; MNP, zinc-containing micronutrient powder, n = 47; PZ, preventive zinc, n = 50) were used to determine the anti-*E. coli* IgG levels. The bars represent the median with the interquartile range of each group and each dot represents an individual sample. The P-value was determined with the Kruskal–Wallis test. (**b**) Changes in the antibody level in each individual. The P-values indicate significant differences between baseline and endline of each intervention group determined by the Wilcoxon matched-pairs signed rank test (**P < 0.01; ***P < 0.001; ns, non-significant). (**c**) The pie charts show the percentages of children who showed no change or an increase or decrease of at least 10% in antibody levels at endline compared with baseline for each group. (**d**) Chi-square analysis of the relative risk and P-values of increasing antibody levels for each intervention group compared with the Control group.

This sub-study was part of a randomised controlled trial and the acute diarrhoea incidence was low (~ 0.6 episodes per 100 days at risk) and similar among the four intervention groups during the study period^20^. Thus, the chance of infection was assumed to be equal across the intervention groups. To examine the effect of zinc intervention on antibody response, the plasma IgG level was determined against pathogenic *E. coli*. Data from all samples were analysed for outliners in the anti-*E. coli* IgG level at baseline. Two samples from the TZ group and three samples from the MNP group were excluded (Supplementary Figure S1). Comparison of plasma anti-*E. coli* IgG levels from all samples showed no significant differences among the groups (P = 0.4998; Fig. 1a and Supplementary Table S1). However, paired-sample analysis revealed significantly increased anti-*E. coli* IgG levels from baseline to endline in the TZ and PZ groups (Fig. 1b). While supplementation with MNP showed a trend for an increase, it did not reach statistical significance (P = 0.0863).

For each group, the percentages of children who showed changes in their anti-*E. coli* IgG levels (an increase or decrease of at least 10% when comparing between baseline and endline) were calculated and are presented in pie charts (Fig. 1c). After the 9-month intervention, more than 30% of children who had received zinc supplementation had increased IgG levels—40%, 34% and 32% of children in TZ, MNP, and PZ groups, respectively – while 22% of children in the Control group had increased IgG levels (Fig. 1c and Supplementary Table S2). The TZ group showed the highest percentage of increased IgG levels while the PZ group showed the lowest percentage of children with decreased IgG levels (2%). However, the Control group showed the lowest percentage of increased IgG. These findings are consistent with the fact that TZ, MNP and PZ were the only groups with significantly increased plasma IgG levels at endline compared with baseline. Statistical analysis of the proportion of samples with increased antibody levels revealed a significant different between the TZ and Control groups (chi-square test, P = 0.029) and a relative risk of 1.80 (Fig. 1d). Moreover, the relative risk for increased antibody levels was 1.55 for MNP and 1.46 for PZ.

### Changes in the avidity index in response to pathogenic *E. coli*

**Figure 2:**
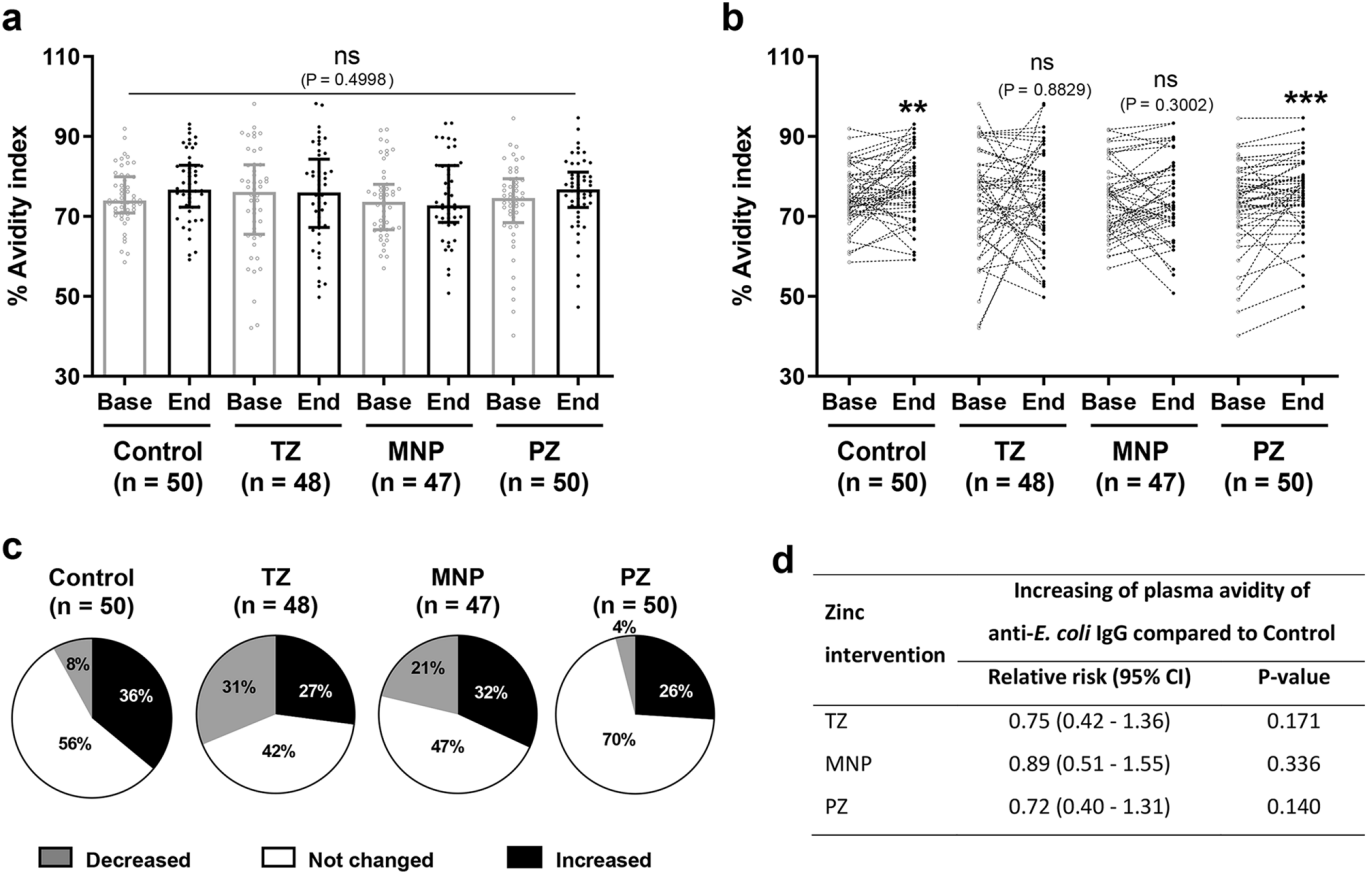
Changes in the avidity index of children among the four intervention groups. (**a**) Plasma samples from baseline and endline of each intervention group (Control, n = 50; TZ, therapeutic zinc with diarrhoea, n = 50; MNP, zinc-containing micronutrient powder, n = 50; PZ, preventive zinc, n = 50) were used to determine the avidity index. Bars represent the median with the interquartile range of each group; each dot represents an individual sample. The P-value was determined with the Kruskal–Wallis test. (**b**) Changes in the avidity index of each individual. The P-values indicate significant differences between baseline and endline of each intervention group determined by the Wilcoxon matched-pairs signed rank test (**P < 0.01; ***P < 0.001; *ns* non-significant). (**c**) The pie charts show the percentages of children who showed no change or an increase or decrease of at least 5% in the avidity index at endline compared with baseline for each group. (**d**) Chi-square analysis of relative risk and P-values of increasing avidity from each intervention group compared with the Control group.

Changes in the avidity index were determined as maturation predictors of the antibody response to pathogenic *E. coli*. Similarly to the antibody levels, the overall avidity index was not different among the groups (P = 0.4998; Fig. 2a). Analysis of paired samples within each group revealed that the avidity index had increased significantly in the Control and PZ groups at endline compared with baseline (Fig. 2b). However, only the PZ group showed a significant increase in both the IgG level and avidity index at endline compared with baseline (Figs. 1b and 2b). For each group, the percentages of children who showed a change in the avidity index (increase or decrease of at least 5% when comparing between endline and baseline) were calculated and are presented in pie chart (Fig. 2c and Supplementary Table S3). The Control group showed 36% of children had an increase in the avidity index and 8% had a decrease in the avidity index. In the PZ group, there were fewer children who show a decrease in the avidity index (4%). There were high percentages of children in the TZ and MNP groups with an increased avidity index: 31% and 21%, respectively. Overall, most children had no changes in their IgG levels and avidity index after the 9-month intervention (Fig. 2b,c). Statistical analysis of the proportion of samples with increased avidity showed no significant difference in any of the treatment groups compared with the Control group, with a relative risk at 0.75 for the TZ group, 0.89 for the MNP group and 0.72 for the PZ group (Fig. 2d).

### Plasma IgG levels and changes in the avidity index in zinc-sufficient or zinc-deficient children

**Figure 3:**
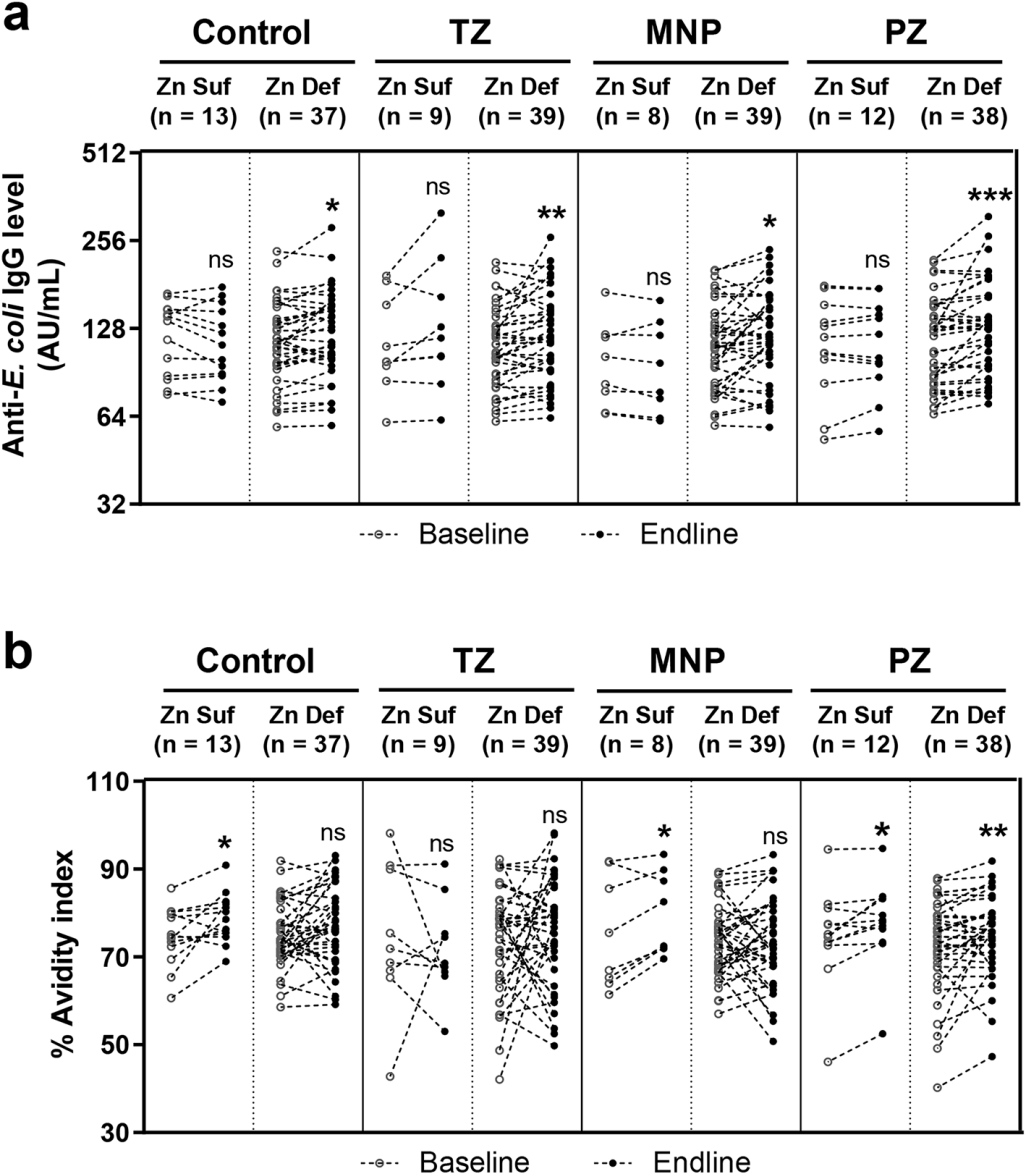
The plasma IgG level and avidity index of the four intervention groups divided by the baseline zinc status. For each group, the (**a**) plasma IgG level and (**b**) avidity index were divided into zinc-sufficient and zinc-deficient sub-groups (*C* control; *TZ* therapeutic zinc with diarrhoea; *MNP* zinc-containing micronutrient powder; *PZ* preventive zinc). Children who had baseline plasma zinc lower than 65 μg/dL were categorised in the zinc-deficient sub-group. The sample sizes are indicated above each graph. Each dot represents an individual sample. *Zn Suf* zinc sufficient group; *Zn Def* zinc deficient group. The P-values indicate significant differences between baseline and endline of each intervention group determined by the Wilcoxon matched-pairs signed rank test. *P < 0.05; **P < 0.01; ***P < 0.001; *ns* non-significant.

More than 79% of children in this study were zinc deficient and the effect of zinc supplementation might be more pronounced in zinc-deficient children. Children in each intervention group were divided into zinc-sufficient and zinc-deficient sub-groups based on the plasma zinc cut-off of 65 μg/dL^24^. At endline, in all intervention groups there were significantly increased IgG levels in children who were zinc deficient. Only zinc-sufficient children in the TZ group showed significantly increased IgG levels (Fig. 3a). Zinc-sufficient children in the Control, MNP and PZ groups showed a significant increase in the avidity index. Only zinc-deficient children in the PZ group showed a significant increase in the avidity index (Fig. 3b). Interestingly, only zinc-deficient children in the PZ group showed significant increases in IgG levels and the avidity index.

## Discussion

The present study determined the IgG levels and avidity index (as a measure of the responsiveness of antibody maturation) of children who received different interventions of zinc supplementation. The different zinc supplementation regimens affected these endpoints. It was especially notable among zinc-deficient children given PZ supplementation, who showed increases in their IgG levels and avidity index. This sub-study of antibody response focussed on the two daily zinc supplementation regimens, PZ and MNP, which both increased plasma zinc and decreased the prevalence of zinc deficiency over the course of the study, as was also reported by the parent study using a slightly larger sample size^19^.

Although zinc supplementation had no effect on cytokine concentrations or T cell counts, interestingly our previous sub-study of immune functions showed that the PZ regimen, given daily as a 7-mg supplement, increased the number of total lymphocytes (perhaps B cells), suggesting further examination of the effect of zinc intervention on these cell subsets or the humoral response to zinc supplementation^17^. Zinc deficiency reduces the B cell response and T cell–dependent antibody responses of B cells^3^. Hence, it makes sense that the children who were zinc deficient at baseline in the PZ group had increases in both IgG levels and the avidity index after the 9-month intervention. From our published data of the parent study at endline, there was a significantly lower prevalence of zinc deficiency in the PZ (60%) and the MNP (67%) groups compared with the TZ (85%) and Control (85%) groups^19^. Moreover, the acute diarrhoea incidence was low and similar among the four intervention groups. A diarrhoea episode lasted for about 2 days, with no group-wise differences in the diarrhoeal duration^20^. These data imply that the results of this study are due to the treatments rather than the exposure to the pathogen. Taken together, these results suggest that intervention by daily preventive zinc tablets is able to increase plasma zinc concentrations and enhance antibody maturation in response to pathogenic bacteria that cause diarrhoea.

In previous studies of the cholera vaccine, children with malnutrition received zinc supplementation for 1 month after vaccination with oral cholera vaccine; they presented enhanced seroconversion of vibriocidal antibodies^26,27^ and cell-mediated immunity in response to cholera^27^. Moreover, a 14-day zinc supplementation during acute shigellosis in children with moderate malnutrition significantly increased antigen-specific antibody levels and lymphocyte proliferation^28^. Furthermore, zinc supplementation as an adjunctive therapy during acute shigellosis significantly improved seroconversion of shigellacidal antibodies and enhanced the number of circulating B lymphocyte subpopulations and plasma cells^29^. These observations are consistent with our results, indicating that daily zinc tablet supplementation improves antibody response and maturation against pathogenic *E. coli*, particularly in zinc-deficient children.

Both the PZ and Control groups showed an increase in the avidity index. Given that avidity maturation is based on hyper-mutation and clonal selection of B cells, maintained and optimal availability of antigen seems to be necessary for complete affinity maturation^30–32^. Lowering the antigenic load through treatment of HIV or tuberculosis infections leads to incomplete avidity maturation^33,34^. However, there were no differences in diarrhoea duration among the four supplementation groups^20^. There are two factors that could affect avidity besides zinc supplementation. (1) Previous infections or exposure (including the number of infections and diarrhoeal duration) of each child, especially to *E. coli*, before being enrolled to the parent study could have affected avidity. (2) Confirmatory bacterial culture from children who had diarrhoea was not performed in the study. The children might be infected with other enteropathogens including *Shigella* spp., *Salmonella* spp., *Vibrio cholerae* and *Campylobacter* spp.^10^. These factors should be considered for future studies. Moreover, the possible reason that some children showed a decrease in the avidity index could be related to *E. coli* exposure during the 9-month intervention. These children might have developed different or new B cell clones^35^, leading to a reduction in avidity in response to intact *E. coli* at endline. However, all supplementation groups had a similar incidence of diarrhoea and the chance of bacterial exposure was assumed to be similar among the groups.

The present study includes samples from an immunology sub-study and the main study, which enrolled a large number of children in a population with a high prevalence of zinc deficiency and close proximity to laboratory facilities suitable for analysis of immune function. Although the outcome of the trial is that a majority of the participants remained zinc deficient at the endline, this present study suggests that taking zinc tablets daily might be the optimal regimen for zinc supplementation based on the increased plasma zinc concentration and enhanced antibody response.

## Methods

### Study design and recruitment

This study used surplus aliquots of plasma samples from a previous sub-study of immune function^17^ nested within a community-based randomised controlled trial examining the optimal zinc supplementation strategy for improving growth, diarrhoeal morbidity and the haematologic and micronutrient statuses in rural Laotian children at risk of zinc deficiency among 3407 children aged 6–23 months^16^. The full study design, method and rationale for the main randomised controlled trial are described by Wessells et al.^16^. Briefly, the previous sub-study aimed to determine the effect of zinc supplementation on immune function in young Laotian children in Khammouane Province^17^. At baseline, non-fasting blood samples of children aged 6–23 months were received at Nakonphanom Hospital, and CBC analysis was performed. Approximately 82% of participants were re-examined at endline after 9 months. The previous analysis of the sub-study included a total of 512 children for whom we had both baseline and endline data. This present study included 200 samples, with 50 samples randomly selected from each of the four intervention groups. These samples had an adequate amount of plasma remaining for the assays performed in the present study.

Children in the parent study were randomised to four intervention groups: (1) daily placebo (Control) powders as the negative control group; (2) 10 days of therapeutic dispersible zinc (TZ) tablets (containing 20 mg zinc) for episodes of diarrhoea; (3) daily preventive multiple micronutrient powders (MNP; containing 10 mg zinc, 6 mg iron, and 13 other vitamin and minerals/day); or (4) daily preventive zinc (PZ) supplements (7 mg zinc/day). The supplementation regimen for the preventive supplements (PZ and MNP) was given daily for 9 months, and caregivers were encouraged to provide therapeutic supplements (TZ) along with ORS starting on the first day of diarrhoea for 10 day for episodes of diarrhoea. According to the intervention protocol^16^, the supplements were given either with or without food.

The trial was approved by the National Ethics Committee for Health Research, Ministry of Health, Lao PDR (NECHR; 040/2014, 069/2015, 039/2016); the University of California, Davis Institutional Review Board (IRB; 626187); and the Khon Kaen University (Thailand) Ethics Committee in Health Research (HE642232). All experiments were performed in accordance with the relevant named guidelines and regulations. Informed consent was obtained from their legal guardians.

### Blood collection and transport

At baseline and endline, venous blood was collected into 7.5-mL lithium heparin tubes (Sarstedt AG & Co; ref. 01.1604.400) for immune assays and plasma zinc measurement, and into a 1.2-mL EDTA tubes (Sarstedt; ref.06.1666.100) for CBC analysis. Plasma separated from 2-mL aliquots of heparinised whole blood was stored at −20 °C prior to laboratory analyses.

### CBC with a five-part differential

This analysis was performed following Nakhonphanom Hospital laboratory protocols as stated previously^7^.

### Plasma zinc analysis

Plasma zinc was analysed by inductively coupled plasma optical emission spectrophotometry (5100 ICP-OES SVDV, Agilent, Santa Clara, CA, USA) at the Children’s Hospital of Oakland Research Institute as described previously^36^.

### IgG antibody measurement

Plasma IgG was detected by ELISA as described by Capelli et al.^37^ Paraformaldehyde-fixed intact pathogenic *E. coli* clinical strain, isolated from clinical samples of 10-month-old girl who had been admitted to Nakhonphanom Hospital, Thailand, was used to coat 96-well polystyrene plate (Nunc, Denmark) at 10^6^ colony-forming units (CFU) with carbonate bicarbonate buffer (pH 9.6) overnight at 4 °C. The plates were washed with washing buffer, 0.1% Tween-20 in phosphate-buffered saline (PBS, pH 7.4), before blocking with 10% foetal bovine serum (FBS) in PBS at room temperature for 2 h. Heparinised plasma samples were diluted at 1:100 with assay diluent (10% FBS in PBS with 0.05% Tween-20). The solution was removed from the pre-coated plates before adding diluted plasma samples and incubated at room temperature for 2 h. After washing, a detection antibody mixture [0.1 μg/mL biotinylated anti-human IgG (BD Biosciences, Franklin Lakes, NJ, USA)], 1:1000 sAv-HRP (BD Biosciences) diluted in assay diluent) was added and incubated for 2 h at room temperature. After washing seven times, 3,3',5,5'-tetramethylbenzidine (TMB) substrate (BD Biosciences) was added and incubated at room temperature for 10 min, before stopping the reaction with 2 N H_2_SO_4_. The absorbance of each well was read at 490 nm by an ELISA reader. The results are shown as AU/mL calculated based on a standard curve of known concentrations of purified human IgG standard^38,39^. The standard curve and quality control chart of each experiment are shown in Supplementary Fig. S2.

The modified protocol to measure the avidity index of plasma IgG was done by duplicate measurements. The protocol involved adding 7 M urea to the samples and incubating them for 1 h prior to the measurement^40,41^. The results are presented as the avidity index (a percentage) calculated with the following formula:

$$\mathrm{Avidity\, index}=\frac{100-\left(\mathrm{IgG \,level\, untreated\, well}-\mathrm{IgG \,level\, treated\, well}\right) \times 100}{\mathrm{IgG \,level untreated \,well}}$$

### Statistical analysis

The normality of the data in each intervention group was tested by the Anderson–Darling test and re-checked for outliners by using a normal Q-Q plot. Outliers at baseline were identified and excluded from the data analysis by using the ROUT method with Q = 10%, the most aggressive setting to remove likely outliners (Supplementary Fig. S1). Differences between the interventions groups were tested with the non-parametric Kruskal–Wallis test. Differences between baseline and endline of each intervention group were tested with the Wilcoxon matched-pairs signed rank test. All analysis was performed using GraphPad Prism 9 (GraphPad Software, San Diego, CA, USA). P ≤ 0.05 was considered to be significant. The power of each test was calculated by post-hoc power analysis with 95% confidence interval and > 80% was acceptable for all experiments.

## Supplementary Information

Supplementary Information.

## Data Availability

The datasets used and/or analysed during the current study are available from the corresponding author on reasonable request.

## Abbreviations

CBC: Complete blood count
Hb: Haemoglobin
MNP: Daily multiple micronutrient powder
PZ: Daily preventive zinc tablets
TZ: Therapeutic dispersible zinc tablets
