# Supplementary material for: Daily preventive zinc supplementation increases the antibody response against pathogenic Escherichia coli in children with zinc insufficiency: a randomised controlled trial
Source: Sci Rep. 2022 Sep 27;12:16084. doi: 10.1038/s41598-022-20445-8 (PMC9515173; doi:10.1038/s41598-022-20445-8)
Supplement: Supplementary file 1 — Supplementary Information. [file 41598_2022_20445_MOESM1_ESM.pdf]

Title: Daily preventive zinc supplementation increases the antibody response against pathogenic *Escherichia coli* in children with zinc insufficiency: a randomised controlled trial

Authors: Chidchamai Kewcharoenwong[1#], Myint Myint Sein[2#], Arnone Nithichanon[3], Aranya Kongmee[2], K Ryan Wessells[4], Guy-Marino Hinnouho[4,5], Maxwell A Barffour[4,6], Sengchanh Kounnavong[7], Sonja Y Hess[4], Charles B Stephensen[4,8], and Ganjana Lertmemongkolchai[1,2]*

Authors' institutions:

[1]Faculty of Associated Medical Sciences, Chiang Mai University, Chiang Mai, Thailand; [2]The Centre for Research & Development of Medical Diagnostic Laboratories, Faculty of Associated Medical Sciences, Khon Kaen University, Khon Kaen, Thailand; [3]Research and Diagnostic Center for Emerging Infectious Diseases (RCEID), Department of Microbiology Faculty of Medicine, Khon Kaen University, Khon Kaen, Thailand; [4]Institute for Global Nutrition, Department of Nutrition, University of California, Davis, CA, USA; [5]Helen Keller International, Washington, DC, USA, [6]College of Health and Human Services, Public Health Program, Missouri State University, Springfield, MO, USA; [7]Lao Tropical and Public Health Institute, Vientiane, Lao People's Democratic Republic; [8]Agricultural Research Service, Western Human Nutrition Research Center, USDA, Davis, CA, USA

***Corresponding authors:** Ganjana Lertmemongkolchai, Faculty of Associated Medical Sciences, Chiang Mai University, Chiang Mai, Thailand, 50200; e-mail: ganjana.l@cmu.ac.th [#]These two authors contributed equally to this work.

Supplementary materials

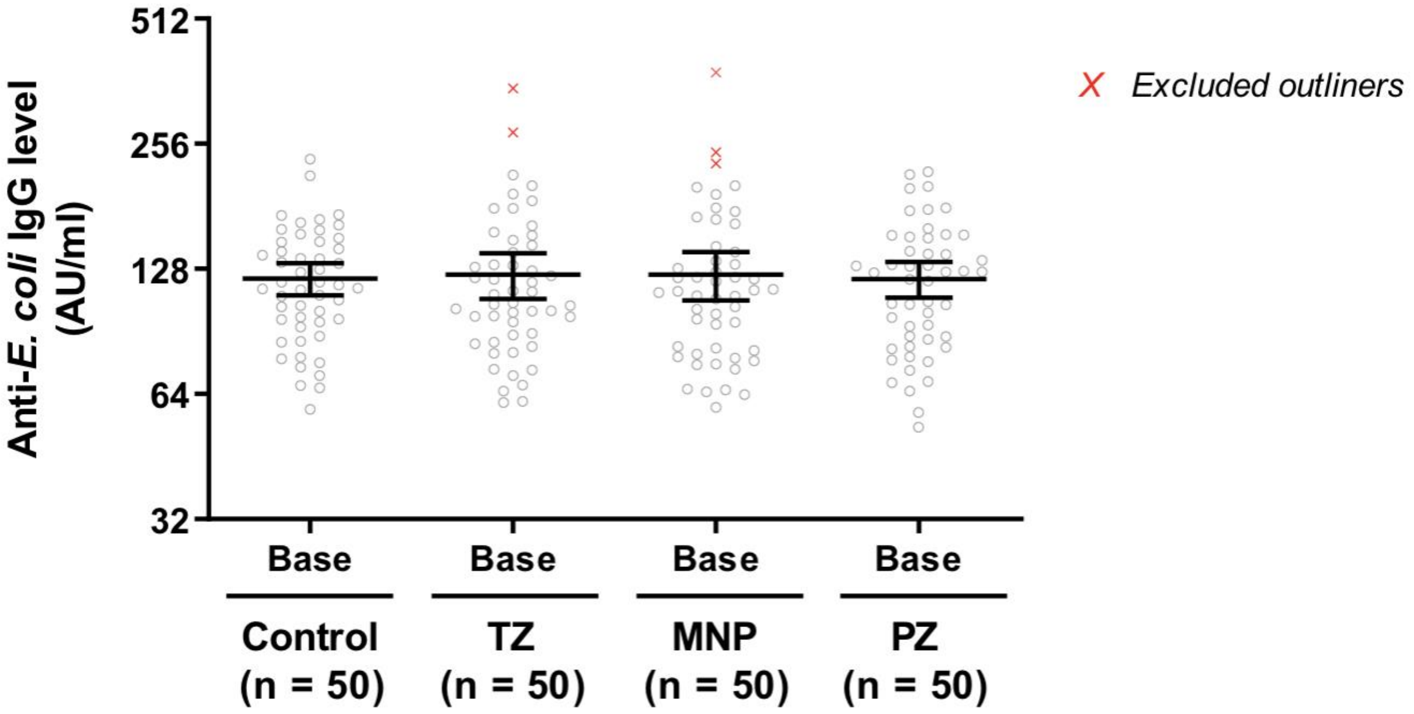

Supplementary Figure S1: Distribution of anti-*Escherichia coli* IgG among the four studied groups. Outliers are shown in red and were excluded from the analysis.

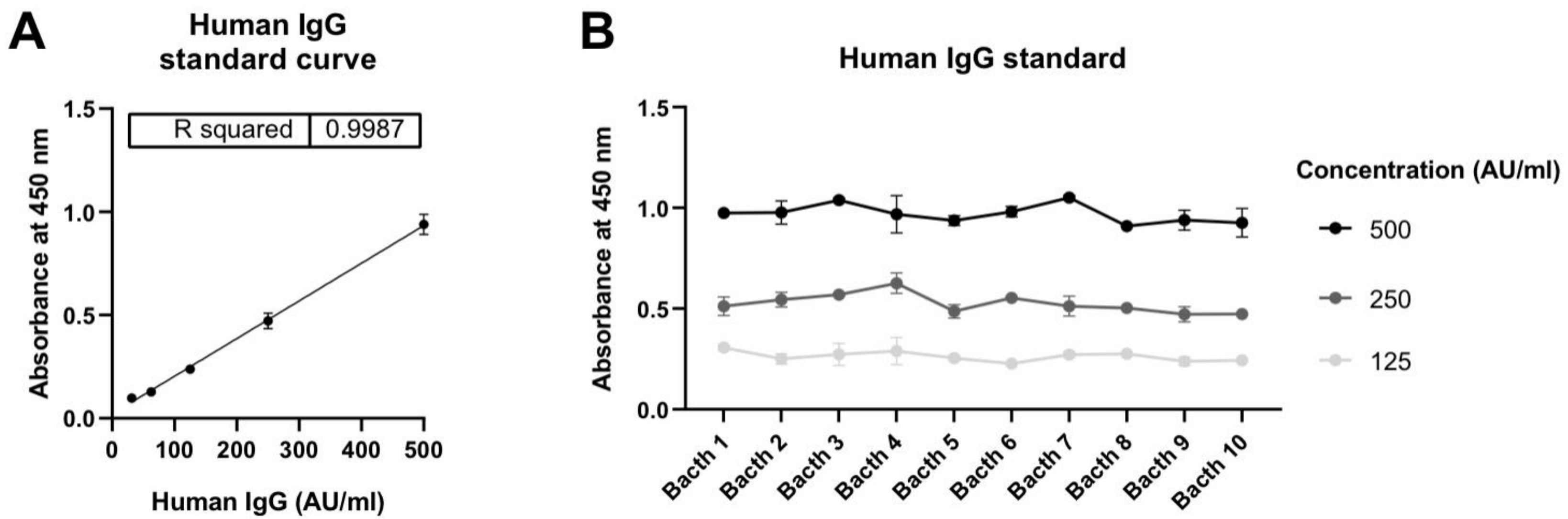

Supplementary Figure S2: Standardisation of human IgG concentration (AU/ml) quantified by enzyme-linked immunosorbent assay (ELISA). Various concentrations of purified human plasma IgG (starting from 50 AU/ml) were coated on ELISA plates. (A) The absorbance of each concentration of the representative plate. (B) Variation in the absorbance of the standard at 500, 250 and 125 AU/ml from each experimental batch.

Supplementary Table S1: Plasma IgG level and the percentages of avidity index in response to pathogenic *E.coli*

Results		All (n=200)	Control (n=50)	TZ (n=50)	MNP (n=50)	PZ (n=50)
**IgG level, AU/ml**	Baseline	122.63 ± 48.70	121.40 ± 37.96	124.04 ± 54.78	123.99 ± 58.11	121.08 ± 41.90
	Endline	133.07 ± 60.14	125.38 ± 43.29	141.04 ± 74.15	133.37 ± 66.40	132.47 ± 52.04
	**P value#**	<0.0001	0.3787	0.0002	0.0215	0.0009
**Avidity index, %**	Baseline	73.37 ± 10.43	74.77 ± 7.15	73.31 ± 13.16	72.51 ± 10.00	72.90 ± 10.81
	Endline	75.42 ± 10.18	77.60 ± 8.42	74.84 ± 12.02	73.53 ± 10.57	75.73 ± 9.34
	**P value#**	<0.0001	0.0080	0.9695	0.1742	0.0019

Values are means ± SDs.

#Variables were compared by Two Way ANOVA between baseline and endline of each intervention group.

Control, placebo; TZ, therapeutic dispersible zinc tablets; MNP, daily multiple micronutrient powder; PZ, daily preventive zinc tablets.

Supplementary Table S2: The number of children whose IgG ratio between endline and baseline were changed more than 10% in response to pathogenic *E.coli*

Results	All (n=200)	Control (n=50)	TZ (n=50)	MNP (n=50)	PZ (n=50)
Increased (> 10%)	66	11	21	18	16
Not changed	114	33	24	24	33
Decreased (> 10%)	20	6	5	8	1

Values are number of children.

Control, placebo; TZ, therapeutic dispersible zinc tablets; MNP, daily multiple micronutrient powder; PZ, daily preventive zinc tablets.

Supplementary Table S3: The number of children whose %avidity index ratio between endline and baseline were changed more than 5% in response to pathogenic *E.coli*

Results	All (n=200)	Control (n=50)	TZ (n=50)	MNP (n=50)	PZ (n=50)
Increased (> 5%)	62	18	14	17	13
Not changed	107	28	21	23	35
Decreased (> 5%)	31	4	15	10	2

Values are number of children.

Control, placebo; TZ, therapeutic dispersible zinc tablets; MNP, daily multiple micronutrient powder; PZ, daily preventive zinc tablets.
